# Supplementary material for: Participation of L-Lactate and Its Receptor HCAR1/GPR81 in Neurovisual Development
Source: Cells. 2021 Jun 30;10(7):1640. doi: 10.3390/cells10071640 (PMC8303161; doi:10.3390/cells10071640)
Supplement: Supplementary file 1 [file cells-10-01640-s001.zip › Supplementary Materials.pdf]

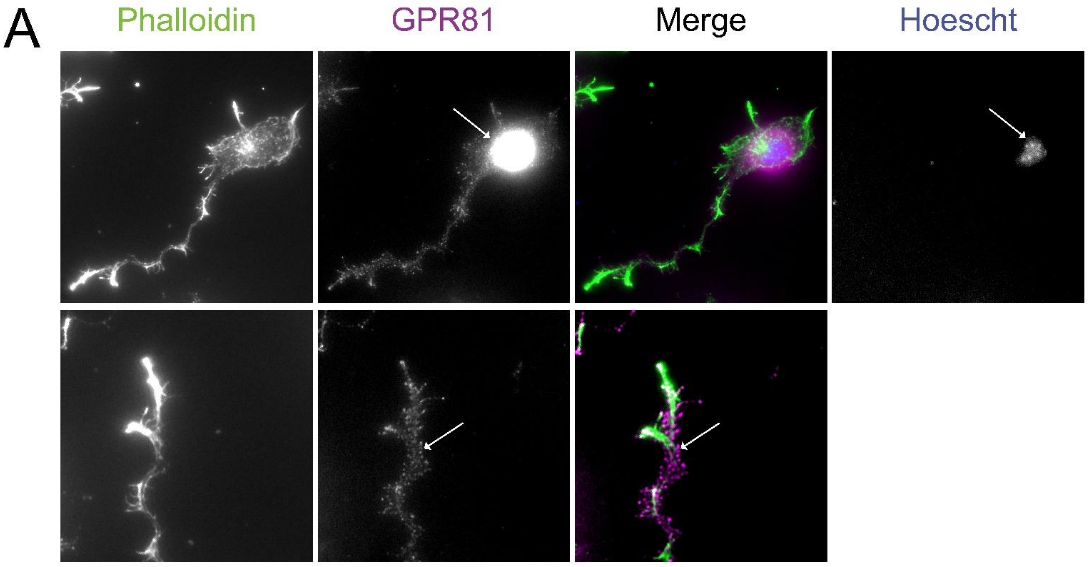

Figure S1. GPR81 is expressed in DIV2 E15 cortical neuron (A) GPR81 is predominantly expressed in the soma, neurite and growth cone. The white arrows indicate colocalization of GPR81 with Hoechst or phalloidin staining.
